# EGFR-TKIs resistance via EGFR-independent signaling pathways

**DOI:** 10.1186/s12943-018-0793-1

**Published:** 2018-02-19

**Authors:** Qian Liu, Shengnan Yu, Weiheng Zhao, Shuang Qin, Qian Chu, Kongming Wu

**Affiliations:** Department of Oncology, Tongji Hospital of Tongji Medical College, Huazhong University of Science and Technology, Wuhan, 430030 China

**Keywords:** EGFR, TKIs, RTKs, ErbB, Drug resistance, Bypass signalings, Downstream compounds

## Abstract

Tyrosine kinase inhibitors (TKIs)-treatments bring significant benefit for patients harboring epidermal growth factor receptor (EGFR) mutations, especially for those with lung cancer. Unfortunately, the majority of these patients ultimately develop to the acquired resistance after a period of treatment. Two central mechanisms are involved in the resistant process: EGFR secondary mutations and bypass signaling activations. In an EGFR-dependent manner, acquired mutations, such as T790 M, interferes the interaction between TKIs and the kinase domain of EGFR. While in an EGFR-independent manner, dysregulation of other receptor tyrosine kinases (RTKs) or abnormal activation of downstream compounds both have compensatory functions against the inhibition of EGFR through triggering phosphatidylinositol 3-kinase (PI3K)/Akt and mitogen-activated protein kinase (MAPK) signaling axes. Nowadays, many clinical trials aiming to overcome and prevent TKIs resistance in various cancers are ongoing or completed. EGFR-TKIs in accompany with the targeted agents for resistance-related factors afford a promising first-line strategy to further clinical application.

## Background

EGFR is a transmembrane glycoprotein belonging to the ErbB family of RTKs which includes ErbB-1 (EGFR), ErbB-2 (HER2/neu), ErbB-3 (HER3), and ErbB-4 (HER4) [1, 2]. Upon binding with ligands, EGFR is activated and leads to the excitation of subsequent intracellular signaling pathways, such as the PI3K/Akt and MAPK, which are involved in the proliferation, differentiation, migration, and apoptosis of certain cells [3–5]. Consequently, overactivation of EGFR signaling pathways is detected in various malignant tumors, including non-small cell lung cancer (NSCLC), breast cancer, head and neck cancer, colon cancer, ovarian cancer, and the like [6–8].

**Fig. 1:**
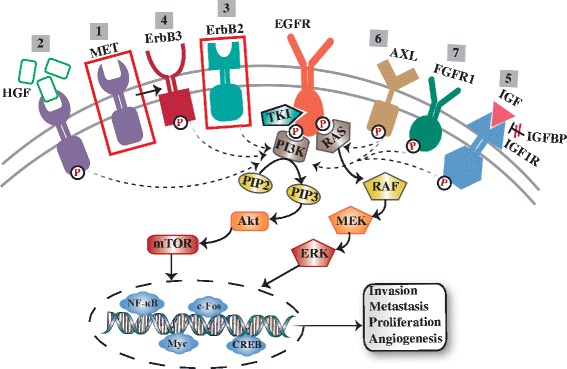
Secondary RTKs-induced EGFR-TKIs resistance. EGFR could trigger downstream PI3K/Akt and MAPK signaling axes which in turn stimulate the transcription factors to drive the associated genes expression which are related with proliferation, angiogenesis, invasion and metastasis. TKIs inhibit EGFR-drived signal transduction by interacting with the tyrosine kinase domain of EGFR. Other RTKs are involved in the development of TKIs resistance via a EGFR-indepenfent way: 1. Amplification of MET activates PI3K through transactivating ErbB3; 2. HGF overexpression; 3. ErbB2 amplification; 4. ErbB3 activation; 5. IGF1R activation by IGF binding or IGFBP reduction; 6. AXL activation; 7. FGFR1 activation

**Fig. 2:**
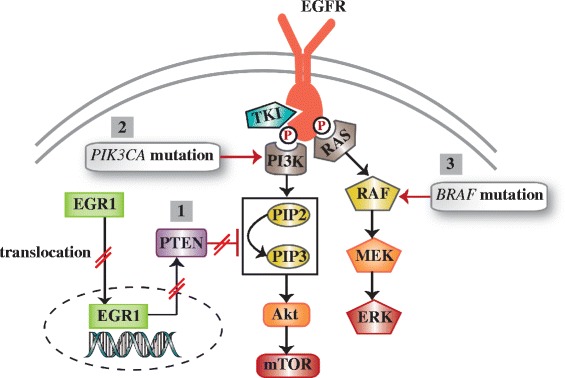
Alternative downstream compounds-induced EGFR-TKIs resistance. 1. PTEN loss: suppressed HGR1 downregulates PTEN expression which in general inhibits the PI3K/Akt activation. 2. *PIK3CA* mutation-drived abnormal activation of PI3K pathway. 3. *BRAF* mutation-drived abnormal activation of MAPK signaling axis

To attenuate the effects that EGFR pathways take on cancers, EGFR TKIs that bind the tyrosine kinase domain of EGFR specifically and inhibit its activity are widely administrated for clinical application. For instance, erlotinib and gefitinib (small molecular EGFR-TKIs) are used to treat patients with EGFR-mutant NSCLC and show significant efficacy [9]. Nevertheless, cancer cells gradually acquire resistance to these drugs, resulting in progression and relapse [10]. Besides the transformation from NSCLC into small cell lung cancer (SCLC) and the process of epithelial to mesenchymal transition (EMT) [11], there are the other two main mechanisms involving in the process of resistance. Firstly, the genetically secondary EGFR mutations could get rid of the inhibition of respective TKIs [12, 13]. Secondly, activation of bypass survival tracks via other RTKs or alternative downstream compounds also account for the acquired resistance [14] (Fig. 1 and Fig. 2). In this review, we mainly focus on the latter mechanism and summarize the existing bypass tracks contributing to TKI resistance via EGFR-independent manners.

## EGFR-triggered signaling pathways in cancers

RTKs are a kind of receptor for various growth factors, cytokines, and hormones. RTKs have a similar molecular structure: an extracellular ligand-binding region, a single hydrophobic transmembrane domain, and a cytoplasmic protein tyrosine kinase region plus additional carboxy terminal and juxtamembrane regulatory regions [3]. The RTK family mainly consists of ErbBs, fibroblast growth factor receptors (FGFRs), insulin-like growth factor receptors (IGFRs), vascular endothelial growth factor receptors (VEGFRs), and hepatocyte growth factor receptors (HGFRs) [3]. Thereinto, EGFR is a paradigm and its intracellular signaling pathways are relevant to the emergence and progression of various cancers, especially NSCLC. Binding with a specific set of ligands, such as epidermal growth factor (EGF), transforming growth factor-alpha (TGF-α), amphiregulin, betacellulin, or epiregulin, EGFR would form a homodimer by itself or form a heterodimer with other ErbB family members. Subsequently, the dimerization of EGFR would activate its cytoplasmic tyrosine kinases domain and then trigger a series of signal transduction [6, 15].

Two primary downstream signaling pathways of EGFR are the PI3K/Akt/PTEN/mTOR and the RAS/RAF/MEK/ERK (Fig. 1). Phosphorylated tyrosine kinase of EGFR acts as a docking site for PI3K which can stimulate the generation of phosphatidylinositol-3,4,5-triphosphate (PIP-3) and promote the activation of Akt [16]. Subsequently, the mammalian target of rapamycin (mTOR), a downstream target of Akt, is activated and provokes the expression of associated proteins needed for the cell cycle progression from the G1 to the S phase [17]. Accordingly, overactivation of this pathway suppresses apoptosis and stimulates tumor growth [18, 19]. Moreover, ligands-EGFR binding drives the MAPK signaling cascade. The dimerization of EGFR activates RAS leading to the phosphorylation of RAF-kinases which in turn phosphorylates MEK. And motivated MEK could impel the activation of ERK inducing to the production of subsequent cell cycle-associated transcription factors (Myc, c-Fos, CREB, NF-κB). And those functional transcription factors ultimately stimulate the cumulation of cyclin D catalyzing the division of cells [20].

## EGFR-independent signaling pathways involved in TKIs resistance

### Secondary RTKs-induced TKIs resistance

#### MET amplification

MET, belonging to the RTKs family, is amplified and relevant to the TKIs resistance in EGFR-dependent cancers, especially in lung cancer. In a gefitinib-sensitive lung cancer cell line HCC827, focal amplification of MET was found to stimulate ErbB3 phosphorylation which in turn activated downstream PI3K/Akt signaling axis compensating the inhibitory effect of gefitinib on EGFR [21]. On the contrary, MET-specific short hairpin RNA (shRNA) restrained MET expression and then recovered the ability of gefitinib to retard PI3K/Akt pathway [21]. Meanwhile, ErbB3-specific shRNA also inhibited the phosphorylation of Akt and controlled the advancement of cell cycle in resistant cells [21]. Moreover, of the 18 gefitinib/erlotinib–resistant lung cancer patients, 4 (22%) with high level of MET were detected [21]. NSCLC patients with classic EGFR-activating mutations were reported to have concomitant MET amplification leading to de novo clinical resistance [22]. Besides lung cancer, MET amplification-drived therapeutic resistance was also reported in other ErbB-dependent cancers, such as colorectal cancer, esophagogastric cancer, ovarian cancer, and so on [23–25].

Referring to the mechanisms of MET amplification in TKI-resistant tumors, it was acknowledged that MET amplification was pre-existed at low frequencies in untreated HCC827 cells and NSCLC patients (approximately 4%) [26], and under the subsequently drug-selective pressure, these cells appeared to be the dominant clones holding MET amplification and led to clinical gefitinib or erlotinib resistance [27]. Nevertheless, the reason why above mechanism has not been reported in other EGFR mutant cell lines and cancers is not clear so far.

Dual targeting of EGFR and MET may provide an effective approach to prevent the development of MET-amplified EGFR TKI–resistant tumors [21]. Currently, several advancing clinical trials are conducted to assess the availability of combining the MET-targeted drugs (MET-TKIs or MET-MAbs) with EGFR TKIs in the treatment of EGFR-mutant tumor with MET-amplification [28, 29].

#### Hepatocyte growth factor (HGF) overexpression

HGF, known as the ligand of MET, is primarily produced by lung cancer cells [30] and stromal cells [31]. The binding between HGF and MET induced various biological effects, such as mitogenic, morphogenic, and antiapoptotic activities [32]. And the complex restored the activation of PI3K/Akt pathway driving the TKI resistance and contributing to the carcinogenesis, proliferation, and metastasis in EGFR-mutant lung cancer [33]. It was reported by Yano, S et al. that unlike the MET amplification, HGF-induced MET activation, acting as a specific mechanism of gefitinib resistance in lung adenocarcinoma harboring EGFR-activating mutations, motivated the PI3K/Akt signaling in an ErbB3-independent manner [34].

HGF is not spontaneously secreted at a detectable level in two gefitinib-sensitive lung adenocarcinoma cell lines (PC-9 and HCC827 cells) [35]. By pretreatment with HGF, these two cell lines were rescued from the gefitinib-induced cell death via a dose-dependent manner that the higher concentration of HGF overcome the cell growth inhibitory effect of gefitinib [34]. Consistently, this phenomenon was also showed in H1975, A431 and HN11 cell lines [27]. In addition, a joint study recruiting 97 tumor specimens from Japanese lung cancer patients with EGFR-mutation reported that HGF overexpression was detected more frequently than other factors (T790 M and MET amplification) in both 23 tumors with acquired resistance (61%) and 45 tumors with intrinsic resistance (29%) [36]. The research implied that HGF might play a crucial role in causing both acquired and intrinsic resistance to EGFR-TKI.

Interestingly, HGF facilitated MET amplification both in vitro and in vivo through upregulating pre-existing MET-amplified clones [27, 37]. Therefore, activation of MET signaling axis, either by amplification or ligand stimulation, is a unique bypass resistance of lung cancer cells to TKI. Simultaneous blockade of the two approaches with EGFR-TKI and HGF-MET antagonists could resist the drug resistance and accelerate the successful treatment for lung cancer patients to the full extent.

#### ErbB2/HER2 amplification

In recent years, there are some inconsistent views concerning the influence of ErbB2 dysregulation on the susceptibility of tumor cells to EGFR-TKIs in NSCLC [38–40]. Traditionally, several preclinical and clinical studies focusing on EGFR-positive (including EGFR mutant, high gene copy number and overexpression) NSCLC patients suggested that increased copy number of ErbB2 gene was susceptible to gefitinib therapy and was correlated with better response rate, disease control rate, and survival preclinical studies reported that gefitinib has a prominent antiproliferative effect on tumors with ErbB2 overexpression [41–43]. Nevertheless, ErbB2 copy number is not the necessary and the unique factor influencing anti-tumor effect of gefitinib in NSCLC patients. A multivariate analysis certified that EGFR mutation, by contrast, is a more crucial factor for beneficial clinical outcomes in gefitinib-treated NSCLC patients than ErbB2 and EGFR copy numbers [44]. Intriguingly, in a current study, ErbB2 amplification was recognized as an unacknowledged mechanism mediating the acquired TKIs resistance of NSCLC with the absence of the EGFR T790 M mutation [45]. Of 26 EGFR-mutant lung adenocarcinoma patients with acquired resistance to gefitinib or erlotinib, 3 (12%) were detected with ErbB2 amplification by FISH analysis [45]. In order to verify the potential correlation, wild-type ErbB2 cDNAs was introduced to the TKI-sensitive cell lines (PC-9 and HCC827) and then the ErbB2 amplification (> 50-fold above baseline) resulted in the resistance to erlotinib [45]. Moreover, under the treatment with erlotinib, inhibition of ErbB2 with small interfering RNAs (siRNAs) impeded the growth of PC-9, HCC827, and H3255 cell lines without EGFR T790 M [45]. Afatinib, a TKI targeting both EGFR and ErbB2, in combined with anti-EGFR antibody could remarkably attenuate the ErbB2 signaling and in turn resumed the sensitivity of lung cancer and colorectal cancer to TKIs in vitro and in vivo [45, 46].

#### ErbB3/HER3 activation

It was elucidated that the resistances to EGFR- or ErbB2-TKIs during the treatment of several malignancies were initiated by ErbB3 [47–50]. ErbB3 is a unique member of ErbB family in that it was regarded as an inactive kinase. However, ErbB3 can be transactivated and transphosphorylated by forming a heterodimers with other ErbB members [51]. Functionally, ErbB3 plays a compensatory role in supplanting the TKIs-inhibited EGFR or ErbB2 to trigger and sustain the activation of typical PI3K/Akt signaling pathway in vitro and in vivo [47]. Unlike the EGFR and ErbB2 motivating the PI3K through the adaptor proteins, ErbB3 could bind the p85 subunit of PI3K to activate PI3K directly, implicating the priority and prevalence of the ErbB3-drived resistance in TKIs-treated tumors [52].

ErbB3-induced drug resistance is primarily mediated by three methods. At first, as mentioned above, MET amplification was known to endow ErbB3 signaling with persistent activation and contribute to the resistance to gefitinib in lung cancer cell lines [21]. Besides, it was demonstrated that the ErbB2-ErbB3 heterodimer was responsible for the stimulation of downstream oncogenic signaling in ErbB2+ breast cancer cells [53]. When the ErbB2 was undermined significantly by TKIs, signaling activities buffering the inhibitory effects of TKIs on ErbB2 were recovered through upregulating the production of ErbB3 and weakening the activity of ErbB3 phosphatase so that lead to the resistance to gefitinib and erlotinib [47]. Third, by binding with its ligand heregulin (HRG) or neuregulin 1 (NRG1), ErbB3 formed a heterodimer with another ErbB receptor. Consequently, the ligand-receptor complex strongly triggered PI3K/Akt axis mediating the resistance to anticancer kinase inhibitors in various cancers [54–56]. For example, among nine HER2-amplified breast cell lines, eight were resistant to the lapatinib by applying ErbB3 ligand NRG1 [56]. And Xia et al. suggested that acquired resistance to lapatinib in the HER2+ breast cancer can be driven by autocrine induction of HRG [57]. On account of above mechanisms, inactivating ErbB3 is identified as an encouraging approach to resist drug resistance [58].

#### IGF1R activation

Activation of IGF1R is another mechanism conferring the acquired resistance against gefitinib to EGFR-amplified and EGFR-mutant cancer cell lines [58]. And the signaling mediated by IGF1R participated in the early stage of TKIs-resistance [59].

In gefitinib-resistant A431 squamous cancer cells, sustained PI3K signaling in the presence of gefitinib was a result of IGF1R-induced signal transduction [60]. Concurrent inhibition of EGFR and IGF1R obstructed the initiation of resistance to gefitinib treatment and reverse the resistant phenotype both in A431 cell line and tumor xenografts [60]. The consistent phenomenon was also found in another gefitinib-resistance cell line model, the head and neck HN11 cells [60]. In the sight of the molecular mechanism, gene expression profiles of the resistant cell line models showed that IGF binding proteins-3 (IGFBP-3) and IGFBP-4, known as negative regulators interfering IGF-IGF1R binding and owning IGF-independent growth inhibition activities, were responsible to the IGF1R-triggered drug resistance [60–62]. The reduction of EGF caused by the EGFR-TKIs treatments downregulated the expression of IGFBP-3 and IGFBP-4. This might lead to the maintenance of IGF1R-induced PI3K/Akt signaling confronting the TKIs-mediated EGFR blockade [60]. Undoubtedly, addition of IGFBP-3 to the A431 cells resensitized the effects of gefitinib and retorted the resistance phenotype [60]. Recently, Zhou et al. pointed out that IGF1R induced acquired resistance of NSCLC cells against EGFR-TKIs mainly via stimulating EMT process triggered by upregulated Snail expression and repressed E-cadherin expression [63].

Albeit above preclinical researches showed the potent correlation between the IGF1R activation and TKIs resistance, there was insufficient study focusing on this trend in clinical patients. It has been reported that the high frequency of IGF1R (39–84%) was detected in patients with various cancers [64–67], however, further study is needed to determine the explicit proportion of high IGF1R expression patients among those having TKIs resistance. To sum up, all these findings provide potential therapeutic targets to surmount TKIs resistance in EGFR-mutant cancers and enhance the efficiency of TKIs treatments.

#### Other bypass RTKs

AXL, a subfamily member of RTKs, is correlated with cell survival, proliferation, metastasis, and phagocytosis [68, 69]. The increased abundance of AXL and its ligand (GAS6) was found in EGFR-TKI resistant NSCLC specimens at the frequency of 20% and 25%, respectively [70]. The aberrant activation of AXL was showed to be required for the development of erlotinib resistance in EGFR-mutant NSCLC models both in vitro and in vivo via Akt, MAPK or NF-κB downstream signaling [70]. What’s more, this process driven by AXL may be correlated with some histological changes, such as EMT [71]. Besides NSCLC, overactivation of AXL was also implicated to the emergence of acquired resistance to imatinib in gastrointestinal stromal tumors and to lapatinib in HER2 positive breast tumor [72, 73]. Inhibition or knockdown of AXL either in the A549 cell line or in a xenograft model exhibited a decreased tumor growth rate and a restored chemosensitivity [74, 75]. Collectively, synthetical treatment combining with representative TKIs and AXL inhibitors to patients with acquired resistance may be a promising strategy to enhance the therapeutic efficacy. Another RTK, FGFR1, formed an autocrine loop with its ligand FGF2 and was identified as an alternative pathway mediating the resistance to EGFR-TKI in a PC-9 cell line model [76]. Meanwhile, inhibition of FGFR1 or FGF2 retarded the growth of resistant PC-9 cells and resensitized the cells to gefitinib-treatment.

### Abnormal activation of downstream compounds

#### Phosphatase and tensin homolog (PTEN) loss

PTEN, acting as a tumor inhibitor, negatively regulates the PI3K/Akt signaling cascade by converting PIP-3 back to PIP-2 [77, 78]. The loss of PTEN decreased erlotinib-induced apoptosis and induced erlotinib-resistance in EGFR-mutant cells via reactivation of Akt and EGFR [79, 80]. In the gefitinib-resistant PC-9 cell line model, reduced PTEN expression was relevant with increased Akt phosphorylation [81]. On the other hand, along with the high PTEN expression, the therapeutic efficacy of gefitinib and erlotinib was restored in the gefitinib-sensitive NSCLC PC-9 cell line. And knockdown of PTEN with siRNA in PC-9 cells contributed to acquired resistance to gefitinib and erlotinib [81]. Retrieval of PTEN expression also enhanced the sensitivity of prostate cancer cells to EGFR inhibition [82]. Furthermore, low expression of PTEN was detected in metastases samples from gefitinib-refractory NSCLC patients [81].

Mechanically, the transcription factor, EGR1, is responsible to the abnormal expression of PTEN. By a nuclear translocation manner, EGR1 played a positive role in regulating PTEN expression [83]. However, this manner was found to be suppressed in resistant cell models and be recovered in the revertant models [81]. It is clear that the expression of PTEN can be controlled by downregulated EGR1 at a transcriptional level.

#### *PIK3CA* and *BRAF* mutations

Mutational activation of the downstream signaling components, such as PI3K/Akt or MEK/ERK, which was independent on the EGFR was identified as a novel mechanism of TKIs resistance [84, 85]. *PIK3CA* gene encodes the catalytic subunit of PI3K and has occasionally mutation in lung cancer [84]. In a vitro study, *PIK3CA* mutation which led to sustained PI3K/Akt signaling conferred the resistance of EGFR-mutant HCC827 cells to gefitinib [86]. Whereafter, Sequist, LV et al. firstly demonstrated *PIK3CA* mutations in 5% EGFR-mutant patients with acquired resistance to EGFR-TKIs [84]. Combining TKI and PI3K inhibitor has been introduced to therapeutic intervention in cancers harboring *PIK3CA* mutations.

Additionally, *BRAF*, known as a member of RAS signaling pathway genes, was reported to be involved in pro-mitogenic activity and acquired resistance to EGFR TKIs in lung cancer and colorectal cancer through activating the MAPK signaling axis [87, 88]. *BRAF* mutations were generally existed in malignant melanoma (30%–40%), whereas it only accounted for approximately 1% of NSCLC [85]. Nevertheless, the small proportion of *BRAF* mutations resulted in negative results (poor prognosis) and provided cognition about mechanisms of acquired resistance to EGFR-TKIs in lung cancer [85].

## Mechanisms of resistance to third generation EGFR-TKIs

Nowadays, the third generation EGFR-TKIs, including osimertinib, rociletinib (CO-1686), HM61713 (BI 1482694), ASP8273, EGF816, and PF-06747775, were widely introduced to replace the first generation EGFR-TKIs to overcome the status of drug resistance [89–92]. A recent clinical trial (NCT02151981) showed that AZD9291 significantly improved objective response rate (ORR) and PFS in T790 M-mutant NSCLC patients who had disease progression on first-line EGFR-TKIs [93]. Subsequently, patients were also resistant to these TKIs after 10 months of treatment, suggesting that additional mechanisms may reduce the efficacy of these inhibitors [13]. In vitro experiment identified three major mutants of EGFR (L718Q, L844 V, and C797S) in resistant cell clones. Among them, C797S mutation was a key factor conferring resistance to the third-generation inhibitors in the existence of del 19 [13].

Moreover, bypass tracts including amplifications of other tyrosine kinases or abnormal activation of downstream compound also mediated the resistance to third-generation TKIs. HER2 and MET amplifications led to poor response to CO-1686 and were detected in patients who had disease progression on CO-1686 or osimertinib treatment [94, 95]. Besides, in an AURA trial, re-biopsy tissues of 4 NSCLC patients with acquired resistance to osimertinib showed different mechanisms of resistance, including FGFR1 amplification, PTEN deletion, MAPK1 and Akt3 overexpression, and SCLC transition [96]. KRAS alteration resulting in increased RAS signaling existed in relapsed biopsy tissues and mutant KRAS transduced cells which were both less sensitive to third generation TKIs [95, 97]. Blocking alternative pathways may provide a promising strategy for improving the drug sensitivity and overcoming the resistance to third generation TKIs.

## Conclusions and perspectives

Currently, the mechanism study on the resistance to EGFR-TKIs has attracted broad attention. There are two major ways involving the initiation and development of resistance to TKI. One is the secondary mutations of EGFR which alter the drug target site of EGFR so that prevent effective interaction with TKIs [9, 98]. Another is activation of bypass tracts via an EGFR-independent manner, such as motivating other RTKs or dysregulating downstream signaling components.

Based on the recognition of above resistant mechanisms, new clinical trials covering phase I-IV are emerging to provide therapeutic interventions adapting for patients with refractory or recurring cancers by inhibiting the alternative pathways [99–101] (Table 1). Some of these trials had favorable results and now are available for clinical application. Moreover, new generation of TKIs are on their way to evade the resistance and enhance the therapeutic efficiency. Further clinical evaluation is required to offer individualized treatments for those specific patients.

**Table 1:** The EGFR-independent mechanisms of EGFR-TKIs resistance and relevant clinical trials

Mechanism	Frequency	Agents	Clinical Trials	Phase	Status	Reference
Secondary RTKs						
MET amplification	5%–22%	Crizotinib	NCT02737501 (NSCLC) NCT00932893 (NSCLC)	III III	Ongoing Has results	[21, 84, 102, 103]
		Tivantinib (ARQ197)	NCT01244191 (NSCLC) NCT01575522 (breast cancer)	III II	Completed Has results	
		Cabozantinib (XL184)	NCT00596648 (NSCLC) NCT01834651 (prostate cancer)	I/II II	Completed Has results	
		Capmatinib (INC280)	NCT01870726 (glioblastoma) NCT03040973 (solid tumors)	I/II IV	Completed Recruiting	
		Onartuzumab (METMab)	NCT01456325 (NSCLC)	III	Completed	
		LY2875358	NCT01874938 (gastric cancer)	II	Completed	
		MSC2156119J	NCT01014936 (solid tumors)	I	Has results	
HGF overexpression	29%–61%	Rilotumumab (AMG102)	NCT01233687 (NSCLC)	I/II	Has results	[36, 104]
ErbB2 amplification	12%–37%	Afatinib	NCT02044380 (NSCLC)	III	Has results	[45, 105]
		Lapatinib	NCT00320385 (breast cancer)	III	Has results	
		Trastuzumab	NCT01419197 (breast cancer) NCT00004883 (NSCLC)	III II	Has results Completed	
ErbB3 activation	17%–52%	MM-121	NCT00994123 (NSCLC)	I/II	Has results	[48, 106]
IGF1R activation	39–84%	Linsitinib (OSI-906)	NCT01533181 (SCLC)	II	Has results	[64–67]
		Figitumumab	NCT00673049 (NSCLC)	III	Has results	
AXL activation	20%	TP-0903	NCT02729298 (solid tumors)	I	Recruiting	[70]
FGFR activation	10%–20%	BGJ398	NCT01928459 (solid tumors)	I	Completed	[107, 108]
Alternative downstream components						
PTEN loss	9%	Ipatasertib	NCT02301988 (breast cancer)	II	Completed	[109]
*PIK3CA* mutation	5%	BYL719	NCT01708161 (solid tumors)	I/II	Completed	[84]
*BRAF* mutation	1%	Dabrafenib	NCT01619774 (melanoma)	II	Has results	[85, 110]

## Abbreviations

EGF: Epidermal growth factor
EGFR: Epidermal growth factor receptor
EMT: Epithelial-mesenchymal transition
FGFRs: Fibroblast growth factor receptors
HGF: Hepatocyte growth factor
HGFRs: Hepatocyte growth factor receptors
HRG: Heregulin
IGFBP-3: IGF binding proteins-3
IGFRs: Insulin-like growth factor receptors
MAPK: Mitogen-activated protein kinase
mTOR: Mammalian target of rapamycin
NRG1: Neuregulin 1
NSCLC: Non-small cell lung cancer
ORR: Objective response rate
PI3K: Phosphatidylinositol 3-kinase
PIP-3: Phosphatidylinositol-3,4,5-triphosphate
PTEN: Phosphatase and tensin homolog
RTKs: Receptor tyrosine kinases
SCLC: Small cell lung cancer
shRNA: Short hairpin RNA
siRNA: Small interfering RNA
TGF-α: Transforming growth factor-alpha
TKIs: Tyrosine kinase inhibitors;
VEGFRs: Vascular endothelial growth factor receptors
